# Characterization of A/H7 influenza virus global antigenic diversity and key determinants in the hemagglutinin globular head mediating A/H7N9 antigenic evolution

**DOI:** 10.1128/mbio.00488-23

**Published:** 2023-08-11

**Authors:** Adinda Kok, Rachel Scheuer, Theo M. Bestebroer, David F. Burke, Samuel H. Wilks, Monique I. Spronken, Dennis de Meulder, Pascal Lexmond, Mark Pronk, Derek J. Smith, Sander Herfst, Ron A. M. Fouchier, Mathilde Richard

**Affiliations:** 1 Department of Viroscience, Erasmus Medical Center, Rotterdam, The Netherlands; 2 Center for Pathogen Evolution, Department of Zoology, University of Cambridge, Cambridge, United Kingdom; St Jude Children's Research Hospital, Memphis, Tennessee, USA

**Keywords:** avian influenza virus, antigenic evolution, hemagglutinin, antigenic cartography

## Abstract

Avian A/H7 influenza viruses are a global threat to animal and human health. These viruses continue to cause outbreaks in poultry and have caused the highest number of reported zoonotic infections to date, highlighting their pandemic threat. Evidence for antigenic diversification of avian A/H7 influenza viruses exists; however, knowledge of the drivers and molecular basis of antigenic evolution of these viruses is limited. Here, antigenic cartography was used to analyze the global antigenic diversity of A/H7 influenza viruses and to determine the molecular basis of antigenic change in A/H7N9 viruses. A phylogenetic tree based on all available A/H7 HA sequences was generated, from which 52 representative, genetically diverse, antigens were selected for antigenic characterization using hemagglutination inhibition assays. The resulting data were used to compute an antigenic map using multidimensional scaling algorithms. High antigenic relatedness was observed between antigens and sera belonging to genetically divergent A/H7 (sub)lineages. The most striking antigenic change relative to the timespan of virus isolation was observed for the A/H7N9 viruses isolated between 2013 and 2019 in China. Amino acid changes at positions 116, 118, 125, 130, 151, and 217 in the hemagglutinin globular head were found to be the main determinants of antigenic evolution between A/H7N9 influenza virus prototypes. The A/H7 antigenic map and knowledge of the molecular determinants of their antigenic evolution will aid pandemic preparedness against A/H7 influenza viruses, specifically regarding the design of novel vaccines and vaccination strategies.

**IMPORTANCE:**

A/H7 avian influenza viruses cause outbreaks in poultry globally, resulting in outbreaks with significant socio-economical impact and zoonotic risks. Occasionally, poultry vaccination programs have been implemented to reduce the burden of these viruses, which might result in an increased immune pressure accelerating antigenic evolution. In fact, evidence for antigenic diversification of A/H7 influenza viruses exists, posing challenges to pandemic preparedness and the design of vaccination strategies efficacious against drifted variants. Here, we performed a comprehensive analysis of the global antigenic diversity of A/H7 influenza viruses and identified the main substitutions in the hemagglutinin responsible for antigenic evolution in A/H7N9 viruses isolated between 2013 and 2019. The A/H7 antigenic map and knowledge of the molecular determinants of their antigenic evolution add value to A/H7 influenza virus surveillance programs, the design of vaccines and vaccination strategies, and pandemic preparedness.

## INTRODUCTION

Influenza A viruses have a major impact on human and animal health worldwide (1). They are categorized into subtypes based on the antigenic properties of their surface glycoproteins hemagglutinin (HA) and neuraminidase (NA) (1). Wild aquatic birds form the original reservoir of influenza A viruses, in which the largest diversity of influenza A virus subtypes—16 HA and 9 NA subtypes—has been detected (1). Influenza A viruses are unique in the broad range of host species they infect, which includes poultry, swine, horses, cats, dogs, marine mammals, and humans (1). This broad host range facilitates cross-species transmission and creates opportunities for zoonoses and pandemics, major threats to human health that may have severe socio-economic consequences. Zoonotic events have been reported for several avian influenza A virus subtypes (2). Most notably, A/H7 influenza viruses have caused 1,675 reported human infections to date, which exceeds the number of reported human infections with viruses of the A/H5 (*n* = 975) and A/H9 (*n* = 113) subtypes (3, 4). Generally, infections with A/H7 influenza viruses in humans result in mild respiratory symptoms and/or conjunctivitis (5). However, infections leading to fatal respiratory failure have been reported as well (4). No sustained human-to-human transmission of A/H7 influenza viruses has been observed to date, yet clusters of human infections have been reported in household settings (6, 7). Given the number and severity of confirmed human infections with A/H7 influenza viruses, they represent a substantial pandemic threat.

A/H7 influenza viruses have been detected across six continents and in combination with all nine NA subtypes identified in avian species (5). Avian influenza viruses are occasionally transmitted from wild aquatic birds to poultry, causing generally subclinical to mild disease, and are then referred to as low pathogenic avian influenza viruses (LPAIVs). However, A/H7 LPAIVs, along with A/H5 LPAIVs, can mutate in poultry to become highly pathogenic avian influenza viruses (HPAIVs). HPAIVs cause severe disease in poultry accompanied by high mortality rates and as a result can have a major socio-economic impact. Based on the geographical separation of wild birds using different migratory flyways, avian influenza viruses, including A/H7 influenza viruses, have evolved into two main genetic lineages: the American lineage and the Eurasian-African-Oceanian lineage (8). Conversions from LPAIV to HPAIV have been observed in viruses from both genetic lineages in various countries across six continents (9), underlining the global challenge posed by A/H7 influenza viruses. Of particular interest are the A/H7N9 influenza viruses which emerged in China in 2013, since these account for the vast majority of the reported A/H7 influenza virus human infections (*n* = 1,568) and reported fatal cases (*n* = 616). Human infections with A/H7N9 viruses mainly occurred between 2013 and 2017 over the course of five epidemic waves (3, 4). The LPAIV A/H7N9 virus converted to HPAIV in early 2017, causing fatal outbreaks in—and the culling of—many gallinaceous birds, after which mass poultry vaccination programs were initiated in China. This led to a substantial decrease in the number of reported A/H7N9 detections in poultry and humans (10). However, A/H7N9 viruses were not fully eradicated, as they have since been detected in poultry (11, 12) and an additional human case was reported in 2019 (13). Zoonotic events with other LPAIV and HPAIV from both A/H7 genetic lineages have been reported as well (5).

Influenza virus surface glycoproteins are known to be subject to antigenic drift, which refers to the gradual accumulation of mutations, allowing immune escape (1). Antigenic drift poses a challenge for influenza virus disease control and vaccination programs. While antigenic drift is a well-studied phenomenon for seasonal human influenza viruses (1), knowledge of the drivers and molecular basis of antigenic evolution of avian influenza viruses is limited. A high degree of antigenic relatedness between genetically diverse A/H7 viruses has been described in several studies on particular subgroups of viruses or limited sets of antigens (14 – 20). Yet, a comprehensive overview of A/H7 antigenic diversity is missing. Such an analysis could provide valuable information aiding A/H7 influenza control in poultry as well as pandemic preparedness. Evidence for antigenic diversification within A/H7 influenza viruses has led to the selection of 12 candidate vaccine viruses (CVVs) by the World Health Organization (WHO) (21, 22), highlighting the challenge the A/H7 subtype poses for pandemic preparedness. An overview of the global A/H7 influenza virus antigenic diversity and evolution could provide a framework for the efficient selection and evaluation of A/H7 CVVs.

In the present study, we generated a phylogenetic tree using all publicly available A/H7 HA sequences, from which a representative set of diverse antigens was selected for antigenic phenotyping using hemagglutination inhibition (HI) and virus neutralization (VN) assays. Cross-HI data were analyzed and visualized using antigenic cartography (23). Additionally, we determined the molecular basis in the HA gene of the antigenic evolution of A/H7N9 influenza viruses isolated in China between 2013 and 2019.

## RESULTS

### Selection of representative hemagglutinins based on a comprehensive A/H7 phylogenetic analysis

**Fig 1:**
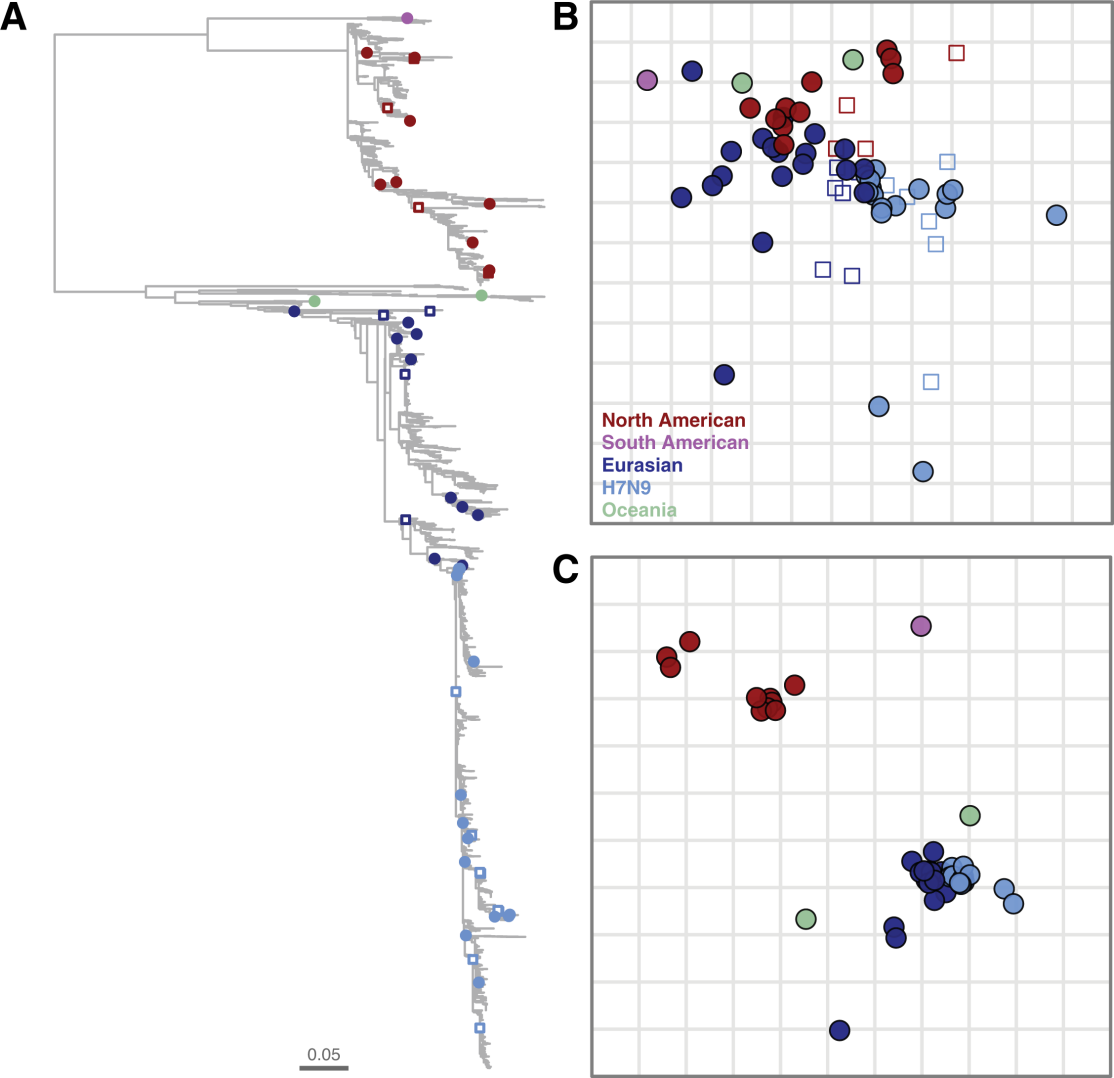
A/H7 influenza virus hemagglutinin phylogenetic tree, antigenic and genetic maps. (**A**) Maximum likelihood phylogenetic tree based on 6560 A/H7 HA1 nucleotide sequences, midpoint rooted. The viruses selected for antigenic characterization are highlighted with closed circles or open squares color-coded based on their respective genetic lineage, North American (red), South American (lilac), Eurasian-African (dark blue), Eurasian A/H7N9 (light blue), and Oceania (light green). Open squares represent viruses against which homologous ferret antisera were raised. The lengths of the branches in the tree are proportional to the number of substitutions per site, according to the branch length scale. A zoomable version of this tree including virus names and bootstrap values is available in Fig. S1. (**B**) Two-dimensional antigenic map constructed from HI titers of 48 A/H7 influenza virus antigens against 16 ferret antisera. Antigens are indicated as closed circles, and antisera are indicated as open squares. Sera and antigens are color-coded based on their respective genetic lineage as described above. The vertical and horizontal directions represent antigenic distance, and one square of the grid corresponds to 1 antigenic unit, which is defined as a two-fold difference in HI titer. An interactive version of this map including virus names is available in Fig. S2A. (**C**) A/H7 genetic map, constructed with the antigens present in the A/H7 antigenic map depicted in (**B**). The genetic map was computed using multi-dimensional scaling algorithms using HA1 amino acids pairwise Hamming distances as input matrix. Antigens are color-coded based on their respective genetic lineage as described above. One square represents a Hamming distance of 10.

All full HA1 A/H7 sequences available through the GISAID (24) and IRD (25) databases were collected. A total of 6,560 HA1 sequences were used to generate a maximum likelihood phylogenetic tree (Fig. 1A; Fig. S1), in which a clear division between the main genetic lineages and sublineages was observed, as described previously (5). Within the American lineage, viruses isolated in North and South America were separated into two distinct genetic sublineages. In the Eurasian-African-Oceanian lineage, Oceanian viruses formed distinct genetic subclades, correlating with their geographical origin (Australia versus New Zealand). In addition, the A/H7N9 viruses which emerged in 2013 in China formed a distinct sublineage. From the phylogenetic tree, 52 genetically diverse antigens were selected for antigenic characterization (Fig. 1A; Table S2), aiming to capture the antigenic diversity of A/H7 HAs. To this end, antigens from different geographical locations, from different outbreaks, from all A/H7N9 infection waves, and genetic outliers were selected.

### Discrepancy between the genetic and antigenic evolution of A/H7 influenza viruses

HA genes from the selected viruses that were not present in-house (Table S2) were ordered synthetically and cloned into a reverse genetics plasmid. Recombinant viruses containing the different H7 HAs and the remaining genes of the A/Puerto Rico/8/1934 (PR/8) were produced by reverse genetics. Ferret antisera were generated against a subset of these viruses based on their genetic and antigenic properties, resulting in a total of 17 antisera in the full data set (Table S2). All antigens were tested against all antisera in HI assays to determine their antigenic properties (Table S3). The data set contained four antigens and one antiserum which had less than three numerical titers, too few to be confidently placed in an antigenic map of two or more dimensions. Two viruses isolated in Mexico, A/chicken/Jalisco/CPA-01861-16-CENASA-95294/2016 and A/chicken/Puebla/CPA-02457-16-CENASA-95294/2016 (26) reacted with only two antisera and with low titers (homologous A/chicken/Puebla/CPA-02457-16C.E.CENASA-95294A/2016 and A/chicken/Karachi/NARC-23B/2003). Moreover, two more recent antigens from the A/H7N9 lineage, A/chicken/Hebei/1009/2020, and A/chicken/Yunnan/1004/2021 (11) reacted with only the serum raised against A/Gansu/23277/2019 (Table S3) and could therefore not be placed confidently in the map. However, the possibility of the latter two viruses being low reactive rather than antigenic variants cannot be excluded without the presence of a homologous antiserum. These four antigens and one homologous antiserum were removed to generate the map. The resulting data set, which contained 48 antigens and 16 sera, was used for the generation of an A/H7 antigenic map using antigenic cartography (Fig. 1B; interactive Fig. S2A that allows visualization of antigen and serum names by hoovering over the points), in which the position of antigens and sera relative to one another is determined using multi-dimensional scaling algorithms such that the distance between them is inversely related to the HI measurement. The validation of the antigenic map is detailed in the Supplementary text, Fig. S3, and interactive Fig. S2B through E.

The antigenic map contained antigens spanning over 40 years of A/H7 influenza virus evolution, from 1979 until 2020. Relatively small antigenic differences were observed between the majority of the antigens. This was exemplified by the fact that half of the antigens were located within an area of three-by-three antigenic units (AUs, 1 AU corresponds to a two-fold difference in the HI assay), including viruses isolated more than 30 years apart. To allow direct comparison between genetic and antigenic properties, we used multi-dimensional scaling to generate a genetic map based on HA1 amino acid Hamming distances between the antigens selected for antigenic characterization (Fig. 1C). In contrast to the antigenic map, a clear distinction between the major genetic lineages and sublineages was observed in the genetic map. Cross-reactivity in HI assays was observed between antisera and antigens from the divergent genetic (sub)lineages (Table S3). This observation was illustrated by the fact that some antigens belonging to different genetic (sub)lineages were closely located to one another in the antigenic map. The discordance between genetic and antigenic characteristics indicates that most genetic changes have little impact on antigenic properties and suggests that the antigenic properties of A/H7 influenza viruses might be governed by only a few amino acid positions in the HA.

In addition to the four viruses which had too few numerical titers to be placed in the antigenic map, the main outliers observed in the antigenic map were the A/H7N9 viruses isolated after the fifth wave of human infections in 2016. Two antigens from 2019, A/Gansu/23277/2019 and A/chicken/Inner_Mongolia/SD010/2019, were located about 6 AU away from the center of the antigenic map (interactive Fig. S2A). Another genetic and antigenic outlier in the data set was the A/Quail/Aichi/5/2009 virus (interactive Fig. S2A), isolated during an outbreak in Japan (27). No A/H7 viruses genetically similar to the viruses isolated during this outbreak have been isolated afterward. Three viruses from the North American sublineage (A/chicken/New-York/SG-00442/2005, A/chicken/New-York/19495-2/2006, and A/chicken/New-York/46545-2/2006), which contained a naturally occurring 24-nucleotide deletion, were located about 2–3 AU away from other viruses from the same sublineage (interactive Fig. S2A).

The WHO CVV antigens, or if not available the genetically closest viruses in our antigenic map (Table S2), are highlighted with larger spheres in Fig. S3J and interactive Fig. S2F. All but four antigens were located within 2 AU of one CVV(-like) antigen, demonstrating that the WHO CVV covered the global antigenic diversity of A/H7 well. Yet, pairs of CVV(-like) antigens were located within 1 AU from one another, suggesting redundancy in the antigenic properties of the WHO CVVs (Fig. S3J, interactive Fig. S2F).

### One to three substitutions in the HA head are responsible for antigenic change in A/H7N9 viruses

**Fig 2:**
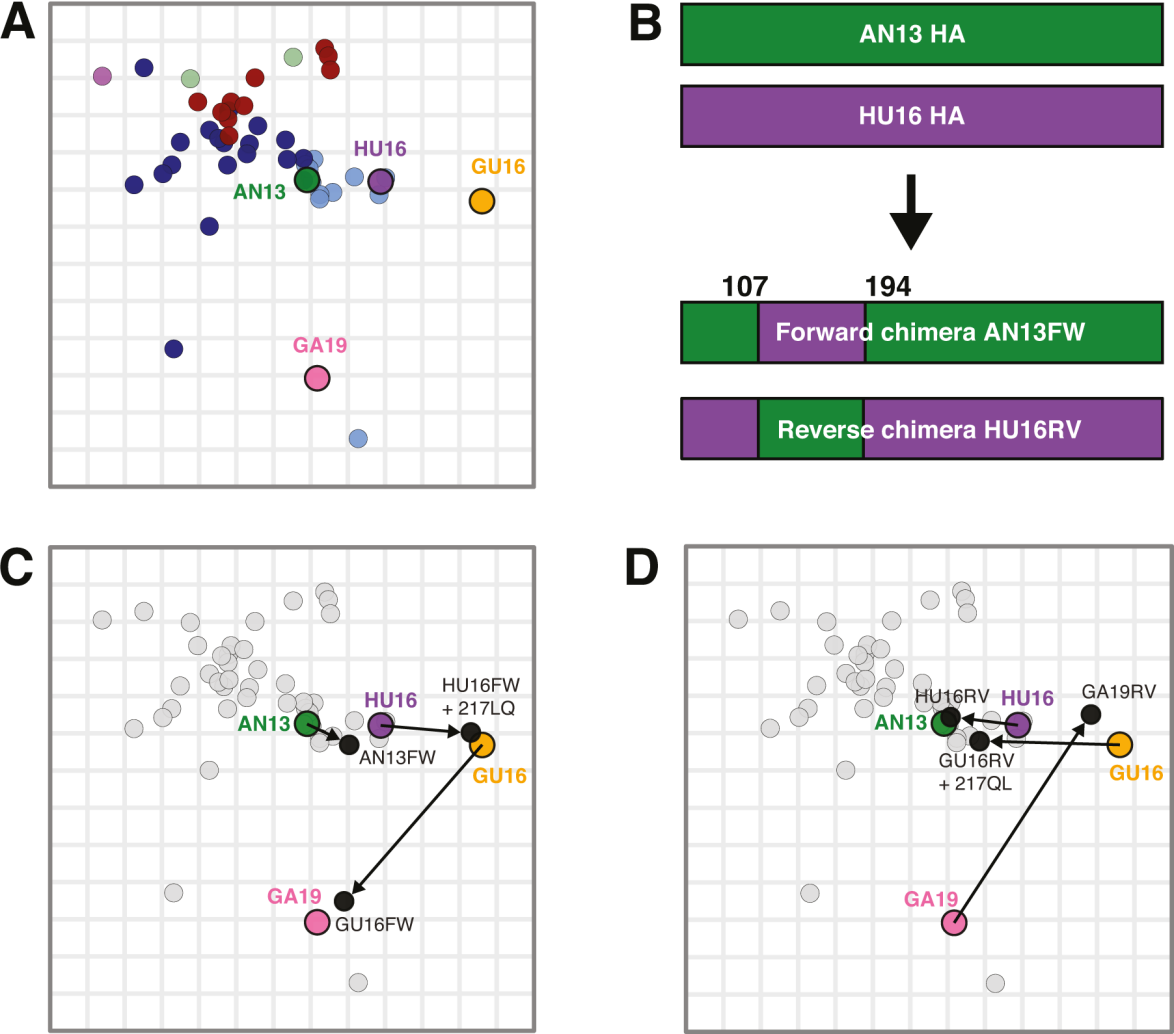
Antigenic diversification over time and antigenic properties of selected A/H7N9 prototypes and chimeric viruses. (**A**) The four selected A/H7N9 prototypes are highlighted as larger closed circles and depicted in different colors in the A/H7 antigenic map: AN/13 (wave 1, green), HU/16 (wave 4, purple), GU/16 (wave 5, yellow), and GA/19 (after wave 5, pink). The rest of the antigenic map is displayed as explained in the legend of Fig. 1. Sera are not displayed. (**B**) Schematic representation of forward and reverse chimeric viruses, with AN13 and HU16 as examples. As depicted, the HA of the forward chimera of AN13 (AN13FW) contains amino acid positions 107–194 of HU16. The reverse chimera for this transition, based on HU16 (HU16RV), contains amino acid positions 107–194 of AN13. Forward chimeric viruses (AN13FW, HU16FW, GU16FW) (**C**) and reverse chimeric viruses (HU16RV, GU16RV, GA19RV) (**D**) are depicted as black closed circles and additional substitutions outside the chimeric region are indicated next to the chimeric virus name, when applicable. The rest of the map is displayed as in (**A**), with the exception that all antigens but the prototypes and mutant viruses are colored in gray. Arrows link prototypes and corresponding chimeric viruses to indicate the effect of the chimeric region and additional substitutions on the antigenic properties of the prototype viruses.

Generally, little antigenic diversity was observed in the period of over 40 years covered in the A/H7 antigenic map. The most striking exception was the Chinese A/H7N9 lineage, for which substantive antigenic drift was observed in only 5 years (Fig. S3K). A/H7N9 viruses isolated during the first waves of human infections were quite similar antigenically; however, those isolated from wave four onwards exhibited different antigenic phenotypes (Fig. S3L). Since these viruses were also responsible for many poultry outbreaks and human infections, we investigated the molecular basis of their antigenic evolution. To this end, four prototypes were selected: A/Anhui/1/2013 (AN13, wave 1), A/Hunan/02650/2016 (HU16, wave 4), A/Guangdong/17SF003/2016 (GU16, wave 5), and A/Gansu/23277/2019 (GA19, after wave 5) (Fig. 2A). The antigenic distances between AN13-HU16, HU16-GU16, and GU16-GA19 were 1.99, 2.77, and 6.50 AU, respectively.

For each of the three transitions, we first generated chimeric viruses to determine the part of the HA in which the amino acids responsible for antigenic change in A/H7 viruses were located. Based on previous observations with the A/H3 (28) and A/H5 (29) subtypes, amino acids 107–194 (A/H7 numbering throughout the manuscript [30]) were swapped between each pair of prototypes, generating forward and reverse chimeric viruses (Fig. 2B). For the AN13-HU16 and the GU16-GA19 transitions, the antigenic properties of the forward (Fig. 2C; Table S3) and reverse (Fig. 2D; Table S3) chimeric viruses corresponded to the prototypes from which amino acids 107–194 were derived. Together, these results indicated that the amino acid(s) responsible for the antigenic differences between AN13 and HU16, and between GU16 and GA19, were located between the positions 107 and 194. In contrast, the forward and reverse chimeric of the HU16–GU16 transition showed similar antigenic properties as HU16 and GU16, respectively (interactive Fig. S4C and D; Table S3), indicating that that the difference in antigenic properties between HU16 and GU16 could not be (solely) attributed to amino acids between 107 and 194. HU16 and GU16 only differed at amino acid position 217 when considering positions located in the HA head, but outside of the chimeric region. Substitution 217LQ and 217QL were introduced in HU16FW and GU16RV respectively, which resulted in a change of antigenic properties of the chimeras to that of GU16 and HU16, respectively (Fig. 2C through D; Table S3).

**FIG 3:**
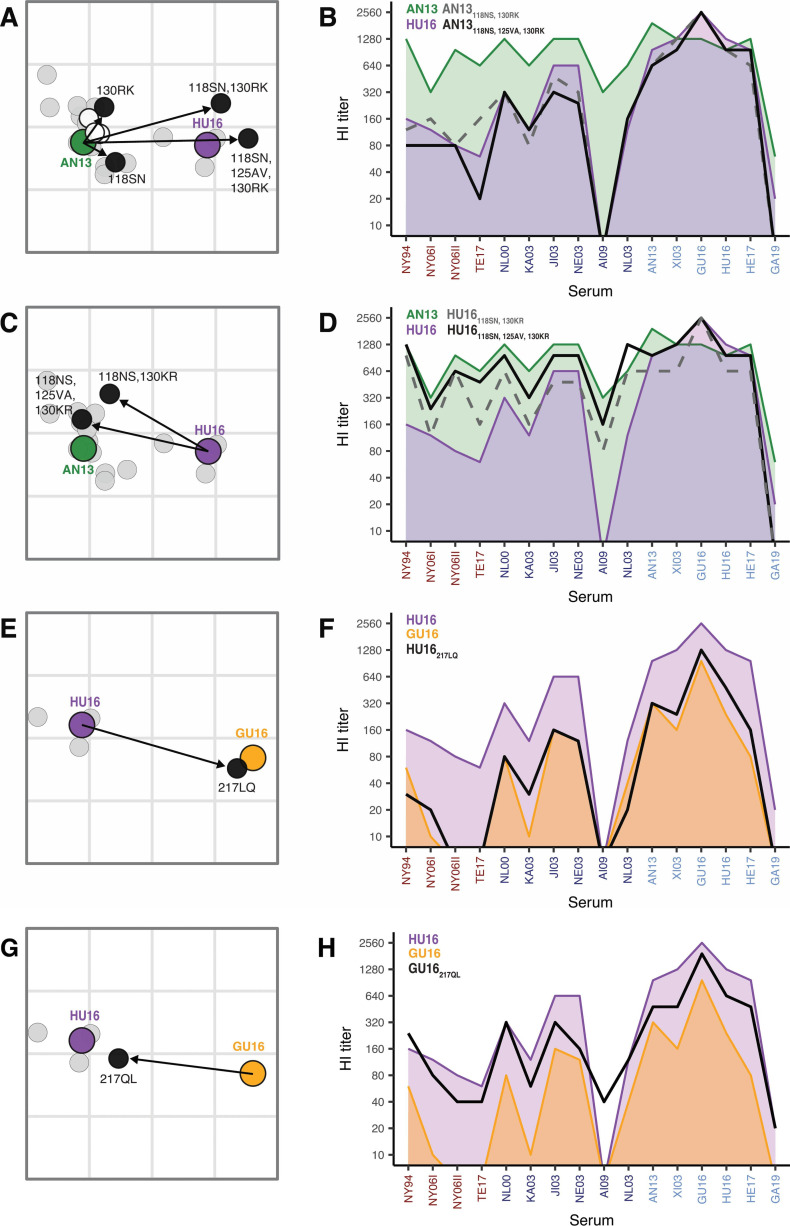

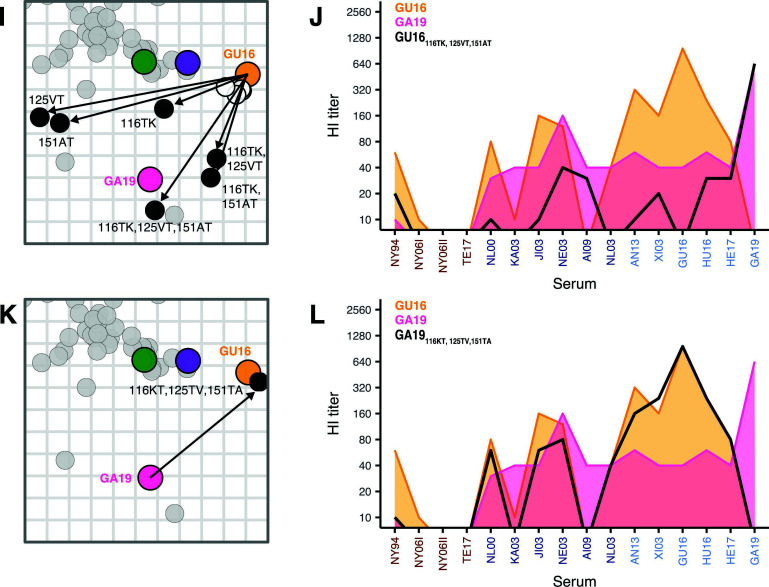
Substitutions in HA mediating the differences in antigenic properties of the A/H7N9 prototypes. Zoomed-in antigenic maps (**A, C, E, G, I, K**) show the effect of single, double, or triple substitutions on the antigenic properties of the A/H7N9 prototypes. Maps are displayed as described in the legend of Fig. 2C and D. In addition, single, double, or triple virus mutants with different antigenic properties as compared to the prototype are colored in black, and the amino acid substitutions present in each mutant virus are indicated. Arrows link prototypes and corresponding mutant viruses to indicate the effect of the introduced substitutions on the antigenic properties of the prototype viruses. Virus mutants that did not show an antigenic effect are colored in white. Parallel coordinate plots (**B, D, F, H, J, L**) show the HI reactivity (y-axis) of prototypes against all ferret antisera (x-axis) in the map as colored areas and that of mutant viruses as solid black or gray dashed lines to visualize their reactivity patterns. (**A–D**) Antigenic differences between AN/13 (wave 1, green) and HU/16 (wave 4, purple). Forward substitutions were introduced in AN/13 (**A, B**) and reverse substitutions in HU/16 (**C, D**). (**E–H**) Antigenic differences between HU/16 (wave 4, purple) and GU/16 (wave 5, yellow). Forward substitutions were introduced in HU/16 (**E, F**) and reverse substitutions in GU/16 (**G, H**). (**I–L**) Antigenic differences between GU/16 (wave 5, yellow) and GA/19 (after wave 5, pink). Forward substitutions were introduced in GU/16 (**I, J**) and reverse substitutions in GA/19 (**K, L**). Interactive visualizations of these data are available in Fig. S4. Sera are abbreviated as follows: NY94, A/CHICKEN/NEW-YORK/SG-00254A/1994; NY06I, A/CHICKEN/NEW-YORK/19495-2A/2006; NY06II, A/CHICKEN/NEW-YORK/19495-2B/2006; TE17, A/CHICKEN/TENNESSEE/17-007147-2A/2017; NL00, A/MALLARD/NETHERLANDS/12C/2000; KA03, A/CHICKEN/KARACHI/NARC-23B/2003; JI03, A/DUCK/JIANGXI/1717B/2003; NE03, A/NETHERLANDS/219E/2003; AI09, A/QUAIL/AICHI/5B/2009; NL03, A/CHICKEN/NETHERLANDS/3D/2003; AN13, A/ANHUI/1D/2013; XI03, A/XINJIANG/98691B/2014; GU16, A/GUANGDONG/17SF003B/2016; HU16, A/HUNAN/02,650A/2016; HE17, A/HENAN/11156B/2017; GA19, A/GANSU/23277B/2019. Viruses are abbreviated as follows: AN13, A/ANHUI/1/2013; HU16, A/HUNAN/02650/2016; GU16, A/GUANGDONG/17SF003/2016; GA19, A/GANSU/23277/2019.

Next, we sought to identify the specific amino acids that determine the antigenic properties of A/H7N9 viruses. For the AN13–HU16 transition, all five amino acid changes between the chimeric regions of two prototype HAs were introduced individually in AN13: 112AT, 118SN, 125AV, 130RK, and 168LI. Amino acid substitutions 118NS and 130RK had the strongest antigenic impact (Fig. 3; interactive Fig. S4A and B; Table S3). Subsequently, these two substitutions were combined in AN13 and the antigenic properties of this forward mutant were very similar to that of HU16 (Fig. 3A and B; interactive Fig. S4A and B; Table S3). To confirm the antigenic impact of 118NS and 130RK, we took the reciprocal approach by generating the corresponding reverse mutant, HU16_118SN, 130KR_. The HU16_118SN, 130KR_ virus had overall lower reactivity than the AN13 prototype and the reverse chimeric virus, and as a result was located 0.97 AU from the AN13 prototype in the antigenic map (Fig. 3C and D; interactive Fig. S4A and B; Table S3). The 125VA substitution had the largest—albeit very small—antigenic impact of the remaining three amino acid differences present in the chimeric part (interactive Fig. S4). The addition of this substitution to AN13_118NS, 130RK_ and HU16_118SN, 130KR_ showed that the 125VA substitution was needed for full antigenic change between AN13 and HU16 (Fig. 3A through D; interactive Fig. S4A and B; Table S3). The antigenic effects of these substitutions were confirmed in VN assays (Fig. S5A and B; Table S3). Together, the results show that the main determinants of antigenic differences between AN13 and HU16 were changes at positions 118 and 130 and that the substitution at position 125 had an accessory role.

For the HU16–GU16 transition, the substitution at position 217 was identified as necessary for the full antigenic change of the chimeric viruses. To determine whether the antigenic difference between HU16 and GU16 was solely due to the 217LQ substitution or to a combination of the 217LQ substitution with substitution(s) in the chimeric part, we generated a forward HU16_217LQ_ and a reverse GU16_217QL_ mutant virus. Interestingly, the antigenic properties of the HU16_217LQ_ virus were virtually identical to that of the GU16 prototype virus (Fig. 3E and F; interactive Fig. S4C and D; Table S3). The reverse GU16_217QL_ virus was less than 1 AU from the HU16 virus prototype, and its pattern of reactivity in HI was similar to that of the reverse chimeric virus and HU16 prototype virus, albeit the height of reactivity was overall slightly lower (Fig. 3G and H; interactive Fig. S4C and D; Table S3). Similar patterns were observed when assessing the antigenic properties of the mutant viruses in VN, yet the impact of the 217LQ substitution in HU16 was less strong in VN than in HI (Fig. S5C and D; Table S3). Based on these observations, we concluded that the substitution at amino acid position 217 is the major determinant of the observed antigenic difference between HU16 and GU16.

For the GU16–GA19, all eight amino acid differences between the chimeric regions of the two prototype HAs, namely 114GR, 116TK, 125VT, 134SP, 151AT, 163KR, 169IV, and 184KR were individually investigated. Substitutions 125VT and 151AT exhibited the largest antigenic effect (9.60 and 8.76 AU from GU16, respectively) (Fig. 3I; interactive Fig. S4E and F), mainly due to the loss of HI titers against sera from early A/H7N9 viruses (Table S3; interactive Fig. S4E and F). However, these viruses still lacked reactivity against the GA19 antisera. Substitution 116TK was the only amino acid change conferring HI reactivity to the GA19 serum (interactive Fig. S4E and F) and was therefore combined with 125VT and 151AT separately. The antigenic properties of the resulting double-mutant viruses were very similar but remained different from that of the GA19 prototype virus (Fig. 3I; interactive Fig. S4E and F; Table S3). Combining all three substitutions increased the HI titer against the GA19 sera comparable to that of the homologous prototype virus. Yet, the reactivity of the triple mutant virus against other sera was lower than that of the GA19 prototype virus and, as a result, the triple mutant virus was located 1.40 AU away from GA19 in the antigenic map (Fig. 3I and J; interactive Fig. S4E and F). Adding 114GR, 134SP, and 184KR individually to the triple mutant GU/16_116TK, 125VT, 151AT_ did not significantly reduce the distance of the mutant to the GA19 prototype (interactive Fig. S4E and F; Table S3). We subsequently introduced the three substitutions at positions 116, 125, and 151 in reverse in GA19, resulting in GA19_116KT, 125TV, 151TA_. This antigen exhibited antigenic properties which were almost identical to GU16 (Fig. 3K and L; interactive Fig. S4E and F; Table S3). The antigenic phenotypes of the forward and reverse triple mutants were confirmed in VN (Fig. S5E and F; Table S3), leading to the conclusion that the antigenic differences between GU16 and GA19 were mainly due to amino acid changes at positions 116, 125, and 151.

**Fig 4:**
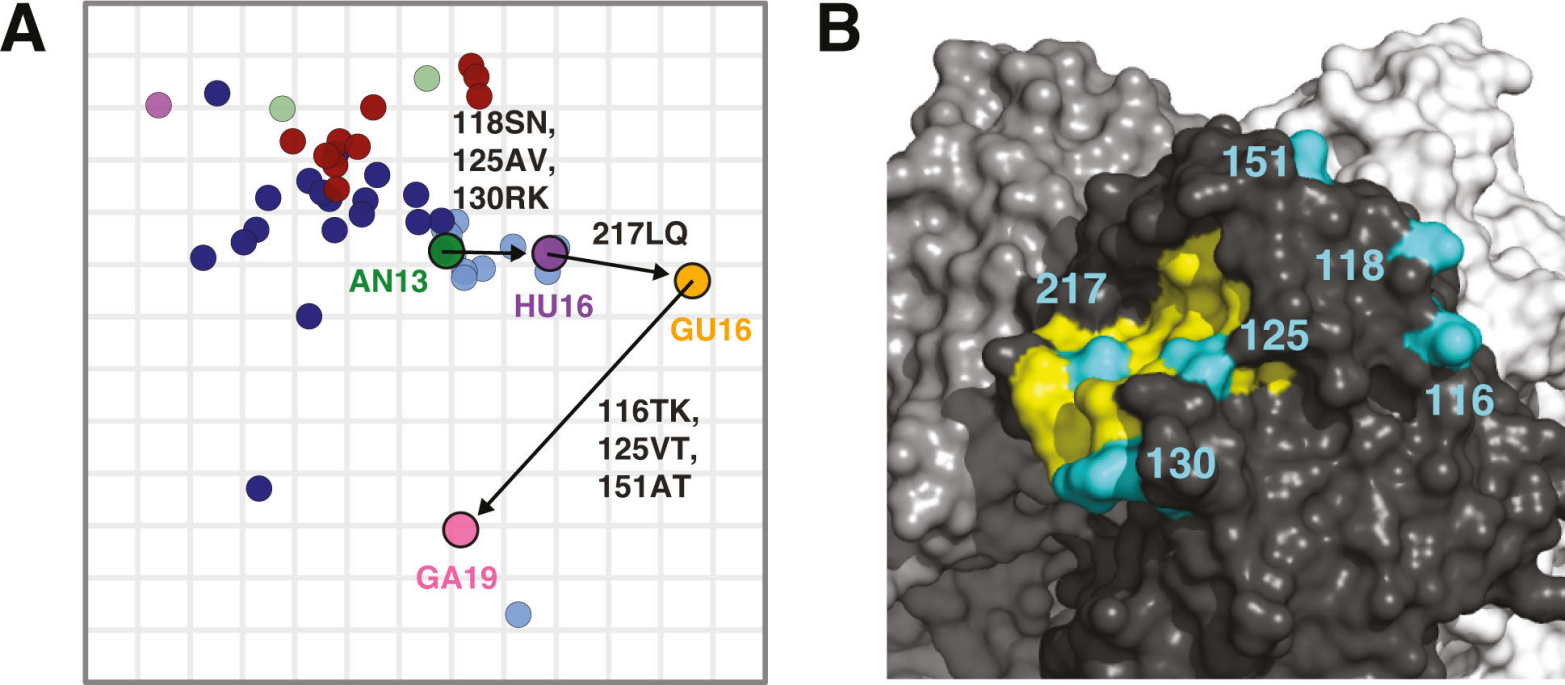
Key substitutions mediating antigenic differences between A/H7N9 prototypes. (**A**) The A/H7 antigenic map is displayed as described in the legend of Fig. 2A. Prototypes are connected by arrows, with the combination of amino acid substitutions mediating antigenic differences indicated. (**B**) Key amino acids determining the antigenic properties of the A/H7N9 viruses displayed on the crystal structure of the A/Anhui/1/2013 HA trimer (PDB 4KOL) (31), visualized with PyMOL (32). The individual monomers are colored in black, gray, and white. On the black monomer, the receptor-binding pocket (33) is indicated in yellow and the amino acid positions of the key substitutions are indicated in light blue.

The molecular basis of antigenic differences between the three studied A/H7N9 prototypes are summarized in Fig. 4. The differences in amino acids at these six positions generally correlated well with the antigenic properties of the antigens in the A/H7 antigenic map (Fig. S6). Mapping the location of the identified amino acids onto the HA trimer structure showed that amino acids 125, 130, and 217 were located directly at the rim of the receptor-binding pocket, whereas amino acids 116, 118, and 151 were located further away from the receptor-binding pocket to the top of the HA globular head (Fig. 4B).

## DISCUSSION

A/H7 influenza viruses are a global threat to animal and human health. In the present study, we quantified and visualized the antigenic diversity of A/H7 influenza viruses and identified amino acids at positions 116, 118, 125, 130, 151, and 217 in the HA globular head to be the main cause of the antigenic evolution of A/H7N9 viruses isolated between 2013 and 2019. Using antigenic cartography, we showed that there was a high antigenic relatedness between antigens and sera belonging to the genetically divergent Eurasian and American lineages, consistent with what others had observed when using a smaller and/or less diverse set of antigens and sera (15 – 20).

Contrary to human influenza viruses which evolve antigenically as a consequence of the buildup of population immunity, the driving forces of the antigenic evolution of avian influenza viruses are less well understood. A/H7 influenza viruses mainly circulate in dabbling ducks and shorebirds, and individual outbreaks of A/H7 viruses in poultry appear to be limited in time and space, despite frequent detection, with the exception of the outbreaks of LPAIV H7N2 in the USA, LPAIV/HPAIV H7N9 in China, and HPAIV H7N3 in Mexico (5). In wild birds, infection with influenza viruses leads to the development of a homosubtypic immunity (34), which can contribute to heterosubtypic immunity (35), which has been hypothesized to be weak and short-lived (36, 37). Nevertheless, several lines of evidence support the fact that immunity in wild birds might be longer lasting than previously thought (38 – 41) and that the dynamics of the detection of avian influenza virus subtypes in wild birds might be driven by the buildup of herd immunity (42). However, intrasubtypic antigenic evolution might be limited by the fact that (i) wild birds can be infected by a large pool of influenza viruses from antigenically distinct subtypes, (ii) influenza viruses from the same subtype can infect a broad range of different bird species, (iii) a large pool of immunologically naive juveniles is present at pre-migratory gatherings which may drive epizootics, and (iv) wild bird populations are separated in time and space. In line with this hypothesis, a study showed that mallards were unlikely to be reinfected with viruses from the same subtypes in the next autumn (43), perhaps limiting the antigenic pressure on one specific subtype. The little antigenic evolution of avian influenza A/H3 viruses in North America is in accordance with this hypothesis (44). In contrast, more intrasubtypic antigenic variation was observed for A/H16 and A/H13 viruses, in which buildup of population immunity might be favored by the fact that these subtypes are primarily found in gulls and terns (45).

Buildup of population immunity in poultry is more likely to be limited than in wild birds, given the short average life span of birds in commercial poultry farms and the fast renewal of the susceptible poultry population, possibly affecting the dynamics of antigenic evolution. In poultry, antigenic evolution is probably mainly driven by poultry vaccination campaigns which have been implemented in several countries to reduce the burden of A/H7 influenza outbreaks, including Pakistan (46), Italy (47, 48), Mexico (49), and China (11). In the latter two countries, the vaccine antigens have been updated over the years in response to the emergence of variants with different antigenic properties (11, 49). Although our study was not designed to specifically address differences in dynamics of antigenic evolution between wild birds and poultry nor the effect of vaccination on antigenic evolution of A/H7 influenza viruses, it is interesting to note that all antigenic outliers, with the exception of A/quail/Aichi/5/2009, were isolated from poultry in countries during or after the implementation of poultry vaccination programs. Two Mexican viruses from 2016, A/chicken/Jalisco/CPA-01861-16-CENASA-95294/2016 and A/chicken/Puebla/CPA-02457-16-CENASA-95294/2016, were not placed in the antigenic map because of too few numerical titers. Both viruses had a low titer against the homologous serum raised against the latter strain. In contrast, a virus isolated early in the outbreak, A/chicken/Jalisco/12283/2012, was antigenically similar to other A/H7 viruses. Three glycosylation sites present in the 2016 Mexican viruses have been hypothesized to be responsible for the change in their antigenic properties, as a result of masking epitopes from antibodies (26) Moreover, two A/H7N9 viruses, A/chicken/Hebei/1009/2020 and A/chicken/Yunnan/1004/2021 displayed large antigenic differences compared to all A/H7 antigens from the map, including GA19 and the vaccine virus that was used from 2019 onwards (A/chicken/Inner-Mongolia/SD010/2019) (50). These findings suggest that the newly isolated A/H7N9 viruses might be able to evade immunity conferred by the 2019 vaccine virus as previously described (11). Interestingly, these viruses contain substitutions at positions 118 and 133, the latter being adjacent to 130 in the HA structure. These observations indirectly support the notion that vaccination programs could result in accelerated antigenic evolution of A/H7 influenza viruses and stress the need for monitoring virus antigenic evolution in response to vaccination to better design poultry vaccination programs and monitor the match with WHO CVVs for pandemic preparedness.

In the present study, we showed that amino acid differences at positions 116, 118, 125, 130, 151, and 217 in the HA globular head were causing the antigenic differences between the A/H7N9 prototypes. Three of these substitutions, 125AT, 151AT, and 217LQ have been identified previously to mediate escape of AN/13 from homologous ferret antiserum (51) and additionally 125AT and 217LQ from human monoclonal antibodies (52). Of note, the 125AT and 151AT substitutions introduce putative *N*-linked glycosylation sites. The 217LQ substitution has previously been described to increase receptor-binding avidity but not specificity, which correlated with decreased sensitivity to inhibition in HI, but not in VN (53). Wang et al. showed that antigenic differences between AN13 and GU16 could only be observed in HI assays but not in VN assays and identified the 217LQ substitution as responsible for these different phenotypes. Here, we observed that the reactivity patterns of AN13 and GU16 were very different in both HI and VN assays. In addition, the antigenic effect of the 217 substitution was also observed in both assays, which is in line with the observations by Chang et al. (52) but in contrast with what Wang et al. observed with mouse and macaque sera (53). The discrepancy between these data could be due to the use of different MDCK cells or the species used for antisera. In addition, the use of antisera against 16 antigens in our study, instead of homologous sera against two antigens, allows for a more detailed characterization of antigenic properties.

The amino acids mediating the antigenic differences between A/H7N9 antigens observed in the present study were located either directly at the rim of the receptor-binding pocket or at the top of the HA globular head, further away from the receptor-binding pocket. This observation contrasts with the findings obtained for human seasonal A/H1, A/H2, A/H3, and B viruses and clade 2.1 A/H5 avian influenza viruses, for which the identified molecular determinants of antigenic variation were immediately adjacent or in very close proximity to the receptor-binding site (28, 29, 54 – 56). Another contrasting observation was that identified substitutions did not necessarily lead to large changes in the biophysical properties of the amino acids, as observed for A/H3N2 viruses (28).

The A/H7 antigenic map will be useful to monitor further antigenic diversification of A/H7 influenza viruses. Moreover, this antigenic map and knowledge of the molecular determinants of antigenic evolution could aid pandemic preparedness against A/H7 influenza viruses, specifically regarding antigen choice and the design of future vaccines. Increased knowledge of the antigenic diversity of influenza viruses that currently circulate in animal reservoirs is vital to assess the breadth of protection required from future vaccines, and surveillance efforts should be intensified to better guide vaccine antigen design and selection processes. Relatively few sequences from Africa, Oceania, and South America were publicly available, creating a geographical bias in the overall data set. Additional data from viruses isolated in these continents would improve the global analysis of the genetic and antigenic diversity of A/H7 influenza viruses. Moreover, given that most antigenic outliers were isolated from poultry in countries during or after the implementation of vaccination programs, increasing surveillance efforts in poultry, especially when vaccination programs are carried out, is warranted to detect antigenic variants. Finally, the knowledge generated here on substitutions that modulate A/H7 antigenic phenotype could be applied to design cross-reactive vaccine antigens, aimed to confer broad reactivity to the majority of viruses in the antigenic space. This antigenic map could serve as a basis for the generation of antibody landscapes (57) to evaluate the breadth of humoral immune responses against a selection of viruses representing the current A/H7 antigenic diversity in (pre-)clinical studies.

## MATERIALS AND METHODS

### Phylogenetic tree construction

All full HA1 A/H7 sequences available through the Global Initiative on Sharing All Influenza Data (GISAID) (24) and Influenza Research Database (IRD) (25) databases on May 25, 2022 were collected. The data set was refined in R using the Biostrings (58) and ape (59) packages. Wrongly annotated non-H7 sequences, identified as sequences for which the average Hamming distance to the whole data set was above 300, were removed. Next, the GISAID sequence entries were complemented with sequence entries unique to the IRD data set based on accession number. The data were screened for duplicate entries based on virus name and/or accession number, which were removed from the data set only if the HA1 sequence was identical. The resulting data set was aligned using MAFFT (60) with the FFT-NS-2 algorithm through a wrapper function from the ips package (61) in R. A maximum likelihood phylogenetic tree (GTR+G+I substitution model, determined with modeltest) was constructed with phangorn (62) in R. Subsequently, 100 bootstrap repeats were performed. The resulting phylogenetic tree was visualized using ggtree (63) in R.

### Cells

293T cells (American Type Culture Collection [ATCC]) were cultured in Dulbecco modified Eagle’s medium (Lonza) supplemented with 10% fetal calf serum (FCS, Sigma-Aldrich), 1× non-essential amino acids (NEAAs, Lonza), 1 mM sodium pyruvate (Gibco), 2 mM L-glutamine (Lonza), and 100 U/mL of both penicillin (PEN) and streptomycin (STR) (Lonza). Madin–Darby canine kidney (MDCK) cells (ATCC) were cultured in Eagle’s minimal essential medium (EMEM, Lonza), supplemented with 10% FCS, 1 × NEAA, 1.5 mg/mL sodium bicarbonate (Lonza), 10 mM HEPES (Lonza), 2 mM L-glutamine, and 100 U/mL of both PEN and STR. Cells were cultured at 37°C, 5% CO_2_, and passaged twice weekly.

### Generation of plasmids and recombinant virus production

If not present in-house yet, synthetic genes containing HA sequences with a monobasic cleavage site were synthesized by Integrated DNA Technologies. These HA genes were subsequently cloned into a previously described modified pHW2000 plasmid (64) by restriction site–based cloning or seamless cloning using the GeneArt Seamless Cloning kit (Thermo Fisher Scientific). Site-directed mutagenesis was performed with the Pfu Ultra II Fusion HS DNA Polymerase (Agilent) and specific primers to remove the multibasic cleavage site (MBCS) when applicable and/or to introduce specific mutations in the HA genes.

Production of recombinant A/H7 influenza viruses was performed by reverse genetics using eight bidirectional plasmids as described previously (64). The A/H7 HA segments of interest were rescued in combination with the remaining seven gene segments of PR/8 or PR/8 high yield (HY) (65). One day prior to transfection, approximately 3 × 10^6^ 293T cells were seeded in gelatin-coated 10-cm dishes. Calcium phosphate–mediated transfection was used to deliver a total of 40 μg of plasmid DNA per dish. Approximately 16 hours after transfection, the cells were washed once with phosphate-buffered saline (PBS) and fresh media containing 2% FCS with 200–350 μg/mL *N*-tosyl-L-phenylalanine chloromethyl ketone (TPCK)-treated trypsin (Sigma-Aldrich) was added. Virus stocks were generated by inoculating either MDCK cells or 11-day-old embryonated chicken eggs with dilutions of the supernatant harvested from the 293T cells 3 days post-transfection. Virus stock production in MDCK cells was performed using EMEM media containing the same supplements as for cell maintenance, but without FCS and with the addition of 20–35 μg/mL TPCK-treated trypsin, referred to as infection media. MDCK supernatants or embryonated egg allantoic fluids were harvested 2–3 days post-inoculation and centrifuged at 1,500 rpm (Allegra X-15R, Beckman Coulter) for 10 minutes to remove debris. The presence of the virus was confirmed by hemagglutination assays using 1% turkey red blood cells (TRBCs) in PBS. Sequences from all plasmids and the HA genes of all virus stocks were confirmed with Sanger sequencing using the BigDye Terminator v3.1 Cycle Sequencing Kit (Applied Biosystems) and the 3500xL Genetic Analyzer (Applied Biosystems).

### Ferret antiserum production

Ferret experiments were performed at the Erasmus Medical Center in Rotterdam, the Netherlands, in strict compliance with the Dutch legislation on the protection of animals used for scientific purposes (2014, European Union directive 2010/63/EU implemented). Experiments were performed under a project license from the Dutch competent authority (license no. AVD101002015340), and the study protocols were approved by the Erasmus Medical Center Animal Welfare Body (permit nos 15-340-04 and 15-340-23). Ferret antisera were generated as described previously (29) in class III isolators under biosafety level 3 conditions. Briefly, male ferrets were inoculated intranasally by applying dropwise 250 μL of recombinant virus carrying the HA (without MBCS) of interest in the PR/8 HY background per nostril. After 14 days, a boost was administered by injecting subcutaneously at two different spots in the back of the ferret a total of 250 μL of concentrated virus combined with 250 μL TiterMax Gold adjuvant (Sigma-Aldrich). Ferrets were terminally bled 14 days after the subcutaneous boost, and antisera were obtained after centrifugation of the blood in VACUETTE 8-mL CAT Serum Separator Clot Activator tubes (Greiner Bio-One) for 15 minutes at 2,000× *g*. Before virus inoculation, subcutaneous boost injection, and the terminal bleed, ferrets were anesthetized with ketamine and medetomidine (antagonized with atipamezole). The concentrated virus used for the subcutaneous boost was prepared by harvesting the allantoic fluids from five embryonated chicken eggs inoculated at 11 days old, which were cleared from debris by centrifuging for 10 minutes at 3,000 rpm (Allegra X-15R). Subsequently, about 36 mL of allantoic fluid was concentrated by centrifuging for 2 hours at 27,000 rpm (SW 32 Ti, Beckman Coulter), and the resulting pellet was resuspended in 700 μL PBS.

### Serological assays

HI assays were performed with recombinant viruses with an HY PR/8 background and virus isolates as described previously (66) using TRBCs. To prevent aspecific inhibition, sera were treated overnight at 37°C with five volumes of an in-house generated *Vibrio cholerae* filtrate containing receptor-destroying enzyme (RDE) to one volume of serum. After inactivation of RDE for 1 hour at 56°C, sera were adsorbed using an equal volume of 10% TRBCs for 1 hour at 4°C to prevent aspecific agglutination. Two-fold serial dilutions of sera in PBS were prepared in round-bottom 96-well plates starting at 1:20 in a volume of 50 μL. To each well, 25 μL of virus diluted in PBS to 4 hemagglutinating units were added. After incubation for 30 minutes at 37°C, 25 μL of 1% TRBC was added to each well and plates were incubated for 1 hour at 4°C before reading the HI titer. The HI titer was determined as the reciprocal value of the highest serum dilution which completely inhibited TRBC agglutination.

VN assays were performed in MDCK cells as described previously (28). First, virus titrations were performed to determine the 50% tissue culture infectious dose (TCID_50_) as described previously (66). Next, sera were incubated for 30 minutes at 56°C to inactivate the complement. Two-fold serial dilutions of sera in PBS, starting at 1:10, were combined with 100 TCID_50_ of virus and incubated for 2 hours at 37°C. Subsequently, serum–virus mixtures were added to flat bottom 96-well plates containing confluent MDCK cells previously washed once with PBS. After incubation for 2 hours at 37°C and 5% CO_2_, cells were washed once with PBS and 200 μL per well of infection media was added. Plates were incubated at 37°C, 5% CO_2_, and the presence or absence of virus in supernatants was determined after 3 days using HA assays with TRBCs. The VN titer was determined as the reciprocal value of the highest serum dilution for which no virus in supernatants was detected. VN assays were performed in duplicate, and the resulting titers were averaged on the log2 scale.

### Genetic and antigenic cartography

Antigenic maps were constructed from HI data using a multidimensional scaling algorithm as described previously (23) using the Racmacs package version 1.1.35 (67) in R. Unless described otherwise, antigenic maps were computed using the “make.acmap” function, with 1,000 optimization runs in two dimensions and the minimum column basis set to zero. The antigenic map was validated using several tests which are described in the supplementary information and in the figure legends corresponding to the display of the test results. For mutant antigens, antigenic maps were computed which contained a single mutant virus in addition to the wild-type virus data set as described above. The average median difference between the mutant maps and the wild-type map was 0.03 AU, thus, the introduction of a single mutant antigen did not significantly alter the wild-type A/H7 antigenic map. The resulting single mutant maps were superimposed on the wild-type antigenic map to generate displays in which one or multiple mutant antigens were visualized using the “mergeMap” function with the frozen overlay method. As such, the positions of mutant antigens were visualized without changing the position of the wild-type antigens nor the position of other mutant antigens in the A/H7 antigenic map. Genetic maps were constructed using a similar approach as described previously (23). HA1 amino acid pairwise Hamming distances were used as the input matrix for non-metric multidimensional scaling algorithms through the function “isoMDS” from the MASS package in R (68). Pairwise distances in the resulting genetic map correlated well with HA1 amino acid Hamming distances (*R* ^2^ = 0.9714).

### Data visualization

Data were visualized with ggplot (69), and interactive plots were generated with plotly (70) in R. The supplementary html files were generated with flexdashboard (71) in R.

## SUPPLEMENTAL MATERIAL

Figure S1 mbio.00488-23-s0001.pdf Maximum likelihood phylogenetic tree of 6560 A/H7 HA1 sequences, midpoint rooted.

Figure S2 mbio.00488-23-s0002.html A/H7 antigenic maps and analysis in interactive html format.

Figure S3 mbio.00488-23-s0003.eps Validation and analysis of the A/H7 antigenic map.

Figure S4 mbio.00488-23-s0004.html Substitutions in HA mediating the differences in antigenic properties between A/H7N9 prototypes.

Figure S5 mbio.00488-23-s0005.eps Virus neutralization data of prototype viruses and final forward and reverse mutants.

Figure S6 mbio.00488-23-s0006.eps A/H7 antigenic maps color-coded by key amino acid changes mediating antigenic differences between A/H7N9 prototypes.

Table S1 mbio.00488-23-s0007.xlsx Overview of the sequences used in the present study, including accession numbers and information on the GISAID contributors of the sequences.

Table S2 mbio.00488-23-s0008.xlsx Overview of A/H7 antigens and post-infection sera used for antigenic characterization.

Table S3 mbio.00488-23-s0009.xlsx HI data used to generate the A/H7 antigenic map displayed in figure 1B, VN data of selected wild-type A/H7 viruses, HI and VN data of A/H7N9 mutant viruses.

Supplemental text mbio.00488-23-s0010.docx Validation of the A/H7 antigenic map.

ASM does not own the copyrights to Supplemental Material that may be linked to, or accessed through, an article. The authors have granted ASM a non-exclusive, world-wide license to publish the Supplemental Material files. Please contact the corresponding author directly for reuse.
