# Supplementary material for: Characterization of A/H7 influenza virus global antigenic diversity and key determinants in the hemagglutinin globular head mediating A/H7N9 antigenic evolution
Source: mBio. 2023 Aug 11;14(5):e00488-23. doi: 10.1128/mbio.00488-23 (PMC10655666; doi:10.1128/mbio.00488-23)
Supplement: Figure S1 — Maximum likelihood phylogenetic tree of 6560 A/H7 HA1 sequences, midpoint rooted. [file mbio.00488-23-s0001.pdf]

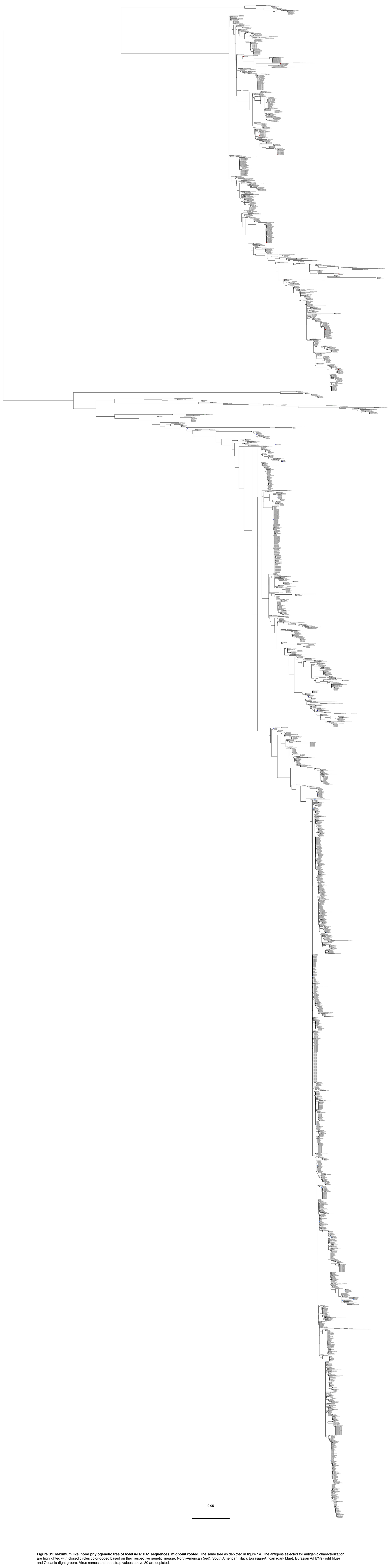

Figure S1: Maximum likelihood phylogenetic tree of 6560 A/H7 HA1 sequences, midpoint rooted. The same tree as depicted in figure 1A. The antigens selected for antigenic characterization are highlighted with closed circles color-coded based on their respective genetic lineage, North-American (red), South American (lilac), Eurasian-African (dark blue), Eurasian A/H7N9 (light blue) and Oceania (light green). Virus names and bootstrap values above 80 are depicted.
