# Supplementary material for: Characterization of A/H7 influenza virus global antigenic diversity and key determinants in the hemagglutinin globular head mediating A/H7N9 antigenic evolution
Source: mBio. 2023 Aug 11;14(5):e00488-23. doi: 10.1128/mbio.00488-23 (PMC10655666; doi:10.1128/mbio.00488-23)
Supplement: Figure S2 — A/H7 antigenic maps and analysis in interactive html format. [file mbio.00488-23-s0002.html]

Figure S2


Figure S2

**Figure S2: A/H7 antigenic maps and analysis in interactive html format.** The interactive html format allows visualization of antigen and serum names by hoovering over the points. On the top right of each panel are different functions to explore the maps. A brief description of each function appears when hoovering over. The total map stress, average stress per titer and average stress per detectable titer are indicated at the bottom left. Interactive maps were made using the ’view’ function in Racmacs. **(A)** The A/H7 antigenic map in two dimensions (2D). This panel shows the same antigenic map in the same visualization as depicted in figure 1B. **(B)** The A/H7 antigenic map in three dimensions (3D). Antigenic map visualization is as described in the legend of figure 1, with the exception that antigens are shown as filled spheres and sera as open cubes. The orientation of the map can be changed by clicking and dragging in the panel. **(C)** Comparison of antigen positions between 2D and 3D antigenic maps. Antigenic map visualization is as described in figure S2B. The colored spheres and cubes show the positions of the antigens and sera in the 3D map, and the connected black filled spheres show the positions of the corresponding antigens or sera in the 2D map. The orientation of the map can be changed by clicking and dragging in the panel. **(D)** A/H7 antigenic map, blobs indicate the area in which a particular antigen or sera can be located without increasing the total map stress by more than one unit. The same map as depicted in figure S3G, where the details of this analysis are described. **(E)** Bootstrap with noise, the same map as depicted in figure S3H. The locations of individual antigen and antisera for each bootstrap repeat can be visualized by clicking on the respective area. The same antigenic map as depicted in figure S3H, where the details of this analysis are described. **(F)** WHO CVVs highlighted as larger spheres in the A/H7 antigenic map, the same map as depicted in figure S3J.

## Column

### A.

### C.

### E.

## Column

### B.

### D.

### F.
