# Supplementary material for: Characterization of A/H7 influenza virus global antigenic diversity and key determinants in the hemagglutinin globular head mediating A/H7N9 antigenic evolution
Source: mBio. 2023 Aug 11;14(5):e00488-23. doi: 10.1128/mbio.00488-23 (PMC10655666; doi:10.1128/mbio.00488-23)
Supplement: Figure S4 — Substitutions in HA mediating the differences in antigenic properties between A/H7N9 prototypes. [file mbio.00488-23-s0004.html]

Figure S4


Figure S4

**Figure S4: Substitutions in HA mediating the differences in antigenic properties between A/H7N9 prototypes.**  Interactive antigenic maps **(A, C, E)** showing the effect of single, double or triple substitutions on the antigenic properties of A/H7N9 prototypes, displayed as described in the legend of figure 3. Antigen and serum names can be visualized by hoovering over the points. Parallel coordinate plots **(B, D, F)** showing the HI reactivity (y-axis) against all ferret antisera (x-axis) in the map as colored areas and that of mutant viruses as solid lines, to visualize their reactivity patterns. Antigen name, serum name and HI titer can be visualized by hoovering over the data points. The display of individual mutants can be disabled by clicking on their name on the right of the plot. **(A, B)** AN13 (wave 1, green) to HU16 (wave 4, purple). **(C, D)** HU16 (wave 4, purple) to GU16 (wave 5, yellow). **(E, F)** GU16 (wave 5, yellow) to GA/19 (after wave 5, pink). Sera are abbreviated as follows: NY94, A/CHICKEN/NEW-YORK/SG-00254A/1994; NY06I, A/CHICKEN/NEW-YORK/19495-2A/2006; NY06II, A/CHICKEN/NEW-YORK/19495-2B/2006; TE17, A/CHICKEN/TENNESSEE/17-007147-2A/2017; NL00, A/MALLARD/NETHERLANDS/12C/2000; KA03, A/CHICKEN/KARACHI/NARC-23B/2003; JI03, A/DUCK/JIANGXI/1717B/2003; NE03, A/NETHERLANDS/219E/2003; AI09, A/QUAIL/AICHI/5B/2009; NL03, A/CHICKEN/NETHERLANDS/3D/2003; AN13, A/ANHUI/1D/2013; XI03, A/XINJIANG/98691B/2014; GU16, A/GUANGDONG/17SF003B/2016; HU16, A/HUNAN/02650A/2016; HE17, A/HENAN/11156B/2017; GA19, A/GANSU/23277B/2019. Viruses are abbreviated as follows: AN13, A/ANHUI/1/2013; HU16, A/HUNAN/02650/2016; GU16, A/GUANGDONG/17SF003/2016; GA19, A/GANSU/23277/2019.

## Column

### A.

### C.

### E.

## Column

### B.

### D.

### F.
