# Supplementary material for: Characterization of A/H7 influenza virus global antigenic diversity and key determinants in the hemagglutinin globular head mediating A/H7N9 antigenic evolution
Source: mBio. 2023 Aug 11;14(5):e00488-23. doi: 10.1128/mbio.00488-23 (PMC10655666; doi:10.1128/mbio.00488-23)
Supplement: Supplemental text — Validation of the A/H7 antigenic map. [file mbio.00488-23-s0010.docx]

**Supplementary text: Validation of the A/H7 antigenic map.**

In order to determine the minimal number of dimensions required to best represent the A/H7 HI data, 100 antigenic maps were generated from 100 random start positions in one to five dimensions. In each repeat, 10% of the data were randomly excluded and were subsequently predicted in the resulting map and the root mean square error (RMSE) between predicted and measured titers was calculated (Fig. S3A). The mean RMSE reduced minimally when comparing maps in two dimensions (mean RMSE of 1.11) to maps in three dimensions (mean RMSE of 1.09). Both 2D (Fig. 1B, interactive Fig. S2A) and 3D (interactive Fig. S2B) antigenic maps represented the HI data well, since good correlations (2D R2 = 0.8388, 3D R2 = 0.8654) between the distances obtained from the HI table and from the map were observed (Fig. S3 B-C). Moreover, the distances between antigens and antisera in the 2D and 3D maps were very comparable (Fig. S3D, R2 = 0.9732). The main differences between the 3D and 2D antigenic map were the positions of two antigens, namely A/duck/Werribee/2563/2007 and A/Guangdong/17SF003/2016 (interactive Fig. S2C, S3E), whose positions in 2D and 3D differed by 3.46 and 5.64 antigenic units (AU), respectively. The stress of these two antigens was also significantly higher in 2D as compared to 3D, whereas for the other antigens these were comparable (Fig. S3F). Since the differences between two and three dimensions were minor, we continued with visualizing the antigenic map in two dimensions for further analysis (Fig. 1B). In order to further verify the robustness of the 2D A/H7 antigenic map, two tests were performed to assess the certainty of antigens and antisera positions. Blobs which indicate the area in which a particular antigen or sera can be located in the antigenic map without increasing the total map stress by more than one unit revealed that the positions of antigens and sera at the periphery of the map were less certain than those located in the center, which is expected given that peripheral antigens and sera show overall lower HI reactivity and are not surrounded by antisera (Fig. S3G, interactive Fig. S2D). In addition, a noisy bootstrap was performed to determine the impact of individual HI measurement error and variability on the position of each antigen and antisera in the antigenic map (Fig. S3H, interactive Fig. S2E). For each of the 1000 bootstraps, normally distributed noise simulating HI measurement error was added to all the HI titers (keeping the average noise per antigen constant) and an antigenic map was computed. Each blob indicates the area where a particular antigen or antisera was located in 68% (equal to one standard deviation in the normal distribution) of the bootstrap. In general, the antigen and serum positions were relatively robust to the added noise, without affecting the topology of the antigenic map and the main conclusions about the antigenic relationships between the antigens and sera (Fig. S3H, interactive Fig. S2E). To determine the impact of individual antigens and sera on the map stability, antigenic maps were generated upon removal of all antigens and sera individually. The average of the median difference in antigen and serum positions between these maps and the map generated with the complete dataset was 0.02 AU when removing individual antigens and 0.11 AU when removing individual sera (data not shown). This indicated that the stability of the A/H7 antigenic map was not strongly depending of the presence of individual antigens and sera. To verify the HI data used to generate the antigenic map, a subset of the antigens was assessed in virus neutralization (VN) assays against all antisera (Table S3). In general, there was a good correspondence between the VN and the HI titers (Fig. S3I), although on average the VN titers were general lower than the HI titers.
